# Bacterial colonization of a power‐driven water flosser during regular use. A proof‐of‐principle study

**DOI:** 10.1002/cre2.393

**Published:** 2021-05-26

**Authors:** Kristina Bertl, Pia Edlund Johansson, Corinna Bruckmann, Matthias Leonhard, Julia R. Davies, Andreas Stavropoulos

**Affiliations:** ^1^ Faculty of Odontology, Department of Periodontology University of Malmö Malmö Sweden; ^2^ Division of Oral Surgery University Clinic of Dentistry, Medical University of Vienna Vienna Austria; ^3^ Division of Conservative Dentistry and Periodontology University Clinic of Dentistry, Medical University of Vienna Vienna Austria; ^4^ Division of Phoniatrics‐Logopedics, Department of Otorhinolaryngology Medical University of Vienna Vienna Austria; ^5^ Faculty of Odontology, Department of Oral Biology University of Malmö Malmö Sweden; ^6^ Division of Regenerative Dentistry and Periodontology University Clinics of Dental Medicine (CUMD), University of Geneva Geneva Switzerland

**Keywords:** AirFloss, biofilm, contamination, interdental cleaning device

## Abstract

**Objectives:**

The present proof‐of‐principle study assessed whether daily use of a power‐driven water flosser (Sonicare AirFloss; SAF) leads to bacterial colonization in the nozzle and/or the device, resulting in contaminated water‐jet.

**Material and Methods:**

In five participants, saliva samples at baseline and water‐jet samples of devices used daily with bottled water for 3 weeks (test) were collected. Additionally, water‐jet samples from devices used daily with bottled water extra‐orally for 3 weeks (positive control) and from brand new devices (negative control), as well as samples from newly opened and 1‐ and 3‐week opened water bottles were collected. Colony forming units (CFU) were recorded after 48 h culturing and 20 oral pathogens were assessed by polymerase chain reaction‐based analysis.

**Results:**

Distinct inter‐individual differences regarding the number of detected bacteria were observed; water‐jet samples of test devices included both aerobic and anaerobic bacterial species, with some similarities to the saliva sample of the user. Water‐jet samples from positive control devices showed limited number of aerobic and anaerobic bacterial species, while the samples from negative control devices did not show any bacterial species. Very few aerobic bacteria were detected only in the 3‐week‐old bottled water samples, while samples of newly and 1‐week opened water bottles did not show any bacterial growth.

**Conclusions:**

The present proof‐of‐principle study showed that daily use of a power‐driven water flosser for 3 weeks resulted in bacterial colonization in the nozzle and/or device with both aerobic and anaerobic, not only oral, species, that are transmitted via the water‐jet.

## 1 INTRODUCTION

Mechanical cleaning of the teeth is essential to minimize the risk of caries and periodontal disease. In addition to the daily use of a manual or electric toothbrush, an interdental cleaning aid should also be used (Chapple et al., 2015; Christou et al., 1998; Noorlin & Watts, 2007; Sälzer et al., 2015; Slot et al., 2008). Several manual (e.g., dental floss, interdental brushes, wooden toothpicks) and a few power‐driven devices (e.g., oral irrigators) are currently available; but based on available evidence no aid can be clearly suggested as superior in terms of effectiveness for plaque removal and/or disease prevention (Slot et al., 2008; Worthington et al., 2019).

Patient acceptability of the method and the degree of patient compliance are important aspects with regards to the effectiveness of the method/device; for example, power‐driven devices seem to be preferred by most patients (Heiß‐Kisielewsky et al., 2015; Shibly et al., 2001). One such power‐driven device for interdental cleaning is the Sonicare AirFloss (Royal Philips N.V., Amsterdam, the Netherlands; SAF), which emits a microburst of high velocity air and liquid micro‐droplets causing a shear stress on the interproximal tooth surface to detach any biofilm accumulation (Rmaile et al., 2014, 2015). Although the clinical efficacy of the SAF is still unclear (Heiß‐Kisielewsky et al., 2015; Mwatha et al., 2017; Stauff et al., 2018), it seems that it indeed achieves higher acceptance among patients compared to flossing (Heiß‐Kisielewsky et al., 2015).

In this context, a possible drawback of such a device is the risk of bacterial colonization and biofilm formation. Since the tip of the nozzle of SAF comes in contact with the oral environment, bacterial colonization of the tip is likely, similarly to toothbrushes (Ankola et al., 2009; Balappanavar et al., 2009; Frazelle & Munro, 2012; Mehta et al., 2007); such colonization might initiate and/or contribute to biofilm formation in the nozzle and/or in the device itself. Furthermore, all water pipework systems are at risk of biofilm build‐up (Gagnon et al., 2005; Wang et al., 2012) and in SAF water is passed from the container of the device to the nozzle and tip through a circuit of channels/pipes inside the device; thus, biofilm formation in SAFs pipework and/or nozzle is likely. Nevertheless, the possibility that viable components of an established biofilm are released from SAF by the water‐jet into the oral cavity has yet not been assessed.

The present proof‐of‐principle study assessed whether daily use of SAF leads to bacterial colonization in the nozzle and/or the device, resulting in a contaminated water‐jet.

## 2 MATERIAL AND METHODS

The present pilot trial was approved by the Swedish Ethical Review Authority (Lund, Sweden; DNR 2014/388) and included five subjects (age range 27–75; three female), with gingivitis or mild periodontitis and insufficient oral hygiene (defined as having a plaque index >20%); patients had not been taking any antibiotics in the last 3 months prior to the study.

### 2.1 Intraoral use of SAF (test)

At baseline, each participant was provided with a brand‐new device and was instructed to use SAF once per day for 3 weeks after tooth‐brushing (either morning or evening) with positioning of the tip of the nozzle from the buccal aspect at each interproximal space. The participants were instructed to use the SAF exclusively with bottled water (one bottle/week; Evian®, Malmö, Sweden), which was provided. Furthermore, the participants were instructed in hygienic use of the device, that is, to rinse the nozzle only with the bottled water, to empty and carefully swab the container with a clean paper tissue after each use, not to touch the tip or nozzle with their fingers, not to drink from the bottled water, not to share the device among family members or with their partners, and not to exchange the nozzle (i.e., the same nozzle was used for 3 weeks). All participants were provided with a bag for transporting the device from and back to the clinic. During the study period the participants were asked not to use any other interdental cleaning device or mouth‐rinse, and also not to receive professional oral hygiene and/or any form of periodontal treatment. After 3 weeks of regular use the participants had to stop using the device for 24 h and then return it to the clinic for water‐jet sampling.

### 2.2 Extra‐oral use of SAF (positive and negative control)

Two brand new devices were used as above for 3 weeks, but extra‐orally (positive controls); that is, bottled water was used (one bottle/week) to fill the container and the device was repeatedly activated until the container was emptied. As above, the device was hygienically used; that is, the nozzle was rinsed only with the bottled water, the container was emptied and swabbed with a clean paper tissue after each use, the nozzle was not touched with the fingers, and the nozzle was not exchanged. Again, the devices were not used 24 h prior to water‐jet sampling. In addition, water‐jet samples from two additional brand‐new devices, filled with bottled water as above, were collected (negative controls).

### 2.3 Saliva, water‐jet, and bottled water sampling

At baseline, a stimulated saliva sample was collected from each participant by using a saliva collection system (Greiner Bio One, Kremsmuenster, Austria) according to the manufacturer's instructions. Specifically, patients were instructed to rinse the oral cavity with the saliva extraction solution (4 ml, citrate buffer pH 4.2) for 2 min; then stimulated whole saliva mixed with the extraction solution was collected in a beaker. For water‐jet sampling, the SAF container was filled with water from a new bottle and 2.5 ml water‐jet samples were collected with the used (test and positive control devices) or a brand‐new nozzle (negative control devices) into a sterile tube. Samples from a newly, and 1‐ and 3‐week opened water bottle were also collected. Immediately after collection and removal of the amount required for culturing, both saliva samples and water‐jet samples were stored at −80°C until polymerase chain reaction (PCR)‐based analysis.

### 2.4 Culturing of aerobic and anaerobic bacteria

The water‐jet samples of the test devices were transferred to brain heart infusion agar plates with blood supplementation and were grown/maintained in 5% CO_2_ in air at 37°C (20 μl water‐jet sample/plate) and anaerobic (100 μl water‐jet sample/plate; 85% nitrogen, 10% hydrogen, 5% CO_2_) conditions. The water‐jet samples of positive and negative control devices, as well as water samples from the newly, and 1‐ and 3‐week opened water bottles were analyzed in a similar fashion, using 200 μl of the water‐jet samples/plate for both conditions. After 48 h, the total number of colony forming units (CFU) per plate was counted and expressed as log_10_ CFU per milliliter sample.

### 2.5 PCR‐based analysis of oral pathogens

All saliva samples, water‐jet samples, and water samples were analyzed by 16S ribosomal RNA‐based PCR with microarray technique (ParoCheck® Kit 20; Greiner Bio‐One, Kremsmuenster, Austria), including the following 20 oral pathogens: *Aggregatibacter actinomycetemcomitans*, *Actinomyces viscosus*, *Tannerella forsythia*, *Campylobacter rectus/showae*, *Treponema denticola*, *Eikenella corrodens*, *Prevotella intermedia*, *Parvimonas micra*, *Porphyromonas gingivalis*, *Fusobacterium nucleatum*, *Actinomyces odontolyticus*, *Capnocytophaga* sp., *Campylobacter concisus*, *Eubacterium nodatum*, *Streptococcus constellatus* group, *Campylobacter gracilis*, *Streptococcus mitis* group, *Prevotella nigrescens*, *Streptococcus gordonii*, and *Veillonella parvula*. The results of the microarray technique were scored semi‐quantitatively as: −, (+), +, ++, or +++.

### 2.6 Scanning electron microscopy

The used nozzle of one randomly chosen participant (#2) was stored in 3% glutaraldehyde after collecting the water‐jet sample. After 24 h, the glutaraldehyde solution was discarded and replaced by a phosphate buffer solution. Then, the sample was dehydrated in a series of ethanol solutions ranging from 70% (v/v) ethanol in distilled water to absolute ethanol, chemically dried with HMDS (Hexamethyldisilazane, Sigma‐Aldrich Life Science, St. Louis), sputtered with gold (Sputter Coater: SC502, Polaron, Fisons Instruments, Surface Science Division, Cambridge, UK) and analyzed with scanning electron microscopy (JSM 6310, JEOL Ltd., Tokyo, Japan).

### 2.7 Statistical analysis

Data derived from culturing and PCR‐based analysis were summarized and reported descriptively.

## 3 RESULTS

All participants returned the SAF after 3 weeks and reported regular daily use of the device as instructed. No discomfort or any other remarkable observation related to SAF use was reported.

### 3.1 Intraoral use of SAF (test) and saliva samples

**FIGURE 1:**
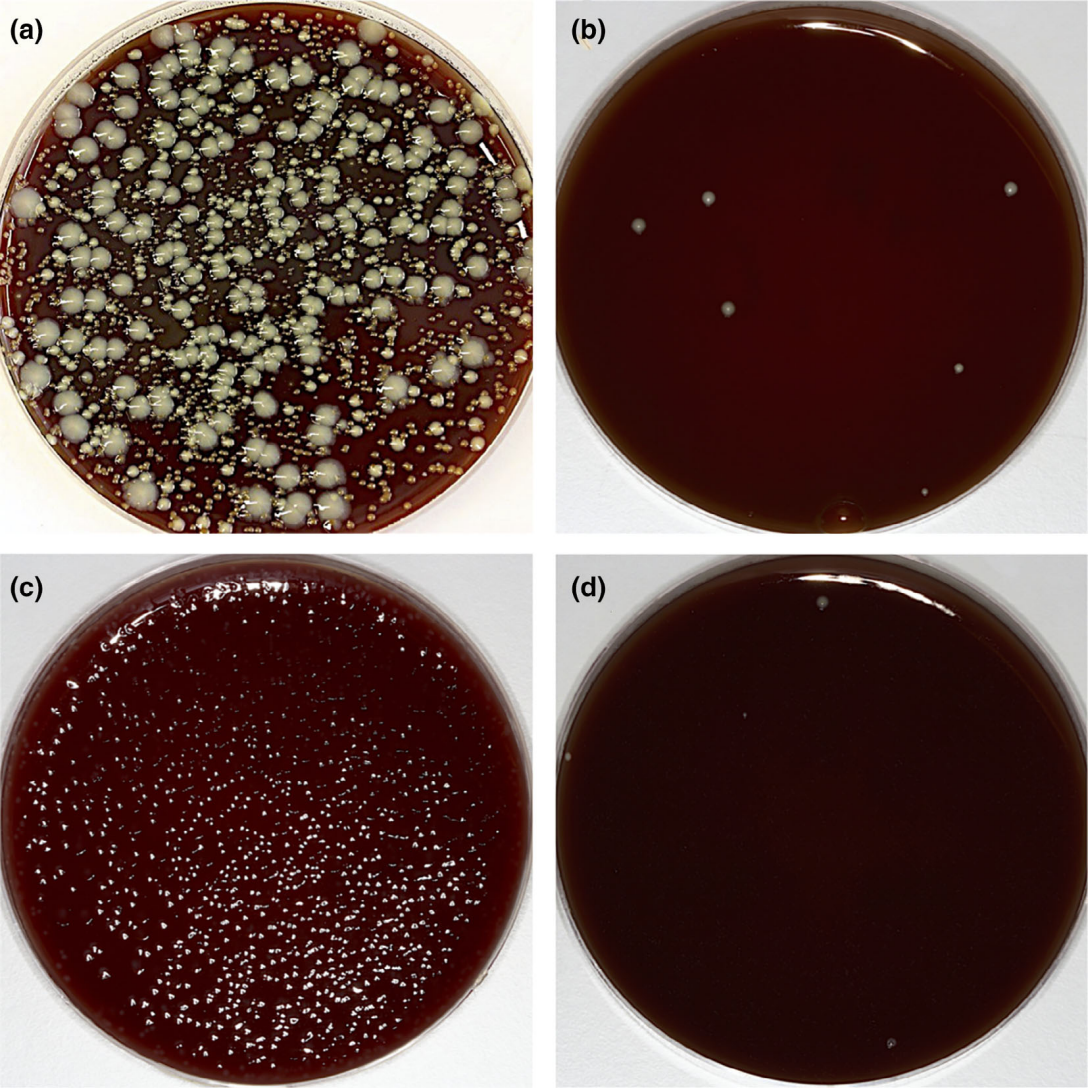
Agar plates after 48 h of culturing with 5% CO_2_ in the air (a, b) and in anaerobic (c, d) conditions. All water‐jet samples, presented aerobic and anaerobic bacterial contamination, typical for water pipes, but clear inter‐individual differences in the number and type of colonies are visible

All water‐jet samples, after 3 weeks of daily intra‐oral use of SAF, presented aerobic and anaerobic bacterial contamination, typical for water pipes; however, inter‐individual differences in the number and type of colonies were present (Table 1 and Figure 1). The total number of log_10_ CFU per milliliter sample ranged for aerobic bacteria from 3.45 to 4.96 (mean 4.33, *SD* 0.62) and for anaerobic bacteria from 2.30 to 4.83 (mean 3.82, *SD* 0.94), respectively. Similar interindividual differences were present in the PCR‐based analysis. While in the water‐jet sample of one participant none of the tested oral pathogens was detected, two participants showed minor contamination with *V*. *parvula* and *S*. *gordonii*, and another two participants showed positive test results for eight oral pathogens (*A*. *viscosus*, *F*. *nucleatum*, *A*. *odontolyticus*, *Capnocytophaga* sp., *S*. *constellatus*, *S*. *mitis*, *S*. *gordonii*, and *V*. *parvula*).

**TABLE 1:** Log_10_ CFU per milliliter sample after 48 h culturing with 5% CO_2_ in the air or in anaerobic conditions

	Log_10_ CFU per ml sample	
	Aerobic bacteria	Anaerobic bacteria
*Intraoral use for 3 weeks (test)*		
#1	3.94	3.73
#2	4.96	4.06
#3	4.58	4.19
#4	3.45	2.30
#5	4.73	4.83
*Extra‐oral use for 3 weeks (positive control)*		
Water‐jet of extra‐orally used SAF #1	3	2.30
Water‐jet of extra‐orally used SAF #2	3.31	2.20
*Single‐time extra‐oral use (negative control)*		
Water‐jet of brand new SAF #1	0	0
Water‐jet of brand new SAF #2	0	0
*Water samples*		
Bottled water	0	0
Bottled water after 1 week	0	0
Bottled water after 3 weeks	1.88	0

Abbreviations: CFU, colony forming unit; SAF, Sonicare AirFloss.

The results of the PCR‐based analysis of the saliva samples is presented in Table 2; 16–19 out of 20 tested pathogens had been tested positive in each sample. Due to the limited number of participants, no correlation between the number and/or type of bacteria detected in the saliva sample and the corresponding water‐jet sample was attempted. Nevertheless, all cases with a positive water‐jet result for one of the oral pathogens also presented a relatively higher number of the specific bacterium in the saliva sample (i.e., the semiquantitative analysis indicated ++ or +++), except for a single case/bacterium (i.e., Participant #2, *A*. *viscosus*; Table 2).

**TABLE 2:** Results of the PCR‐based analysis of 20 oral pathogens

	Intra‐oral use for 3 weeks (test)									
	#1		#2		#3		#4		#5	
	*Saliva*	*SAF*	*Saliva*	*SAF*	*Saliva*	*SAF*	*Saliva*	*SAF*	*Saliva*	*SAF*
*A*. *actinomycetemcomitans*	++	−	+	−	++	−	−	−	++	−
*A*. *viscosus*	++	++	+	++	++	−	++	−	++	−
*T*. *forsythia*	(+)	−	(+)	−	+	−	++	−	+++	−
*C*. *rectus/showae*	+	−	(+)	−	(+)	−	+	−	++	−
*T*. *denticola*	++	−	+	−	++	−	++	−	+++	−
*E*. *corrodens*	+	−	+++	−	(+)	−	++	−	+++	−
*P*. *intermedia*	++	−	++	−	++	−	++	−	+++	−
*P*. *micra*	++	−	−	−	++	−	+++	−	+++	−
*P*. *gingivalis*	−	−	−	−	−	−	−	−	+++	−
*F*. *nucleatum*	+++	+	+++	++	+++	−	+++	−	+++	−
*A*. *odontolyticus*	+++	+	+++	++	+++	−	+++	−	+++	−
*Capnocytophaga* sp.	++	++	++	+++	++	−	++	−	+++	−
*C*. *concisus*	+++	−	++	−	++	−	++	−	−	−
*E*. *nodatum*	−	−	−	−	−	−	−	−	+	−
*S*. *constellatus* group	+++	+	++	(+)	++	−	+++	−	+++	−
*C*. *gracilis*	++	−	−	−	+	−	++	−	+	−
*S*. *mitis* group	+++	(+)	+++	++	+++	−	+++	−	+++	−
*P*. *nigrescens*	++	−	(+)	−	−	−	++	−	+	−
*S*. *gordonii* group	+++	+	++	(+)	+++	−	+++	−	+++	+
*V*. *parvula*	+++	++	+++	+++	+++	−	+++	+	+++	++

Abbreviation: SAF, Sonicare AirFloss.

### 3.2 Extra‐oral use of SAF (positive and negative control) and bottled water samples

After 3 weeks of daily extra‐oral use of SAF (positive control), all water‐jet samples presented aerobic and anaerobic bacterial contamination, however, the number was distinctively lower compared to water‐jet samples from the test SAF devices (Table 1). No bacterial growth was observed in water‐jet samples from negative control devices. Similarly, no bacterial growth was observed in samples from a newly opened and 1‐week‐old water bottle. Very few aerobic bacteria were detected in samples from the 3‐week‐old bottled water. None of the water jet‐samples of the positive and negative controls, nor any of the water samples showed any positive results in the PCR‐based analysis of oral pathogens.

### 3.3 Biofilm formation at the tip of the nozzle

**FIGURE 2:**
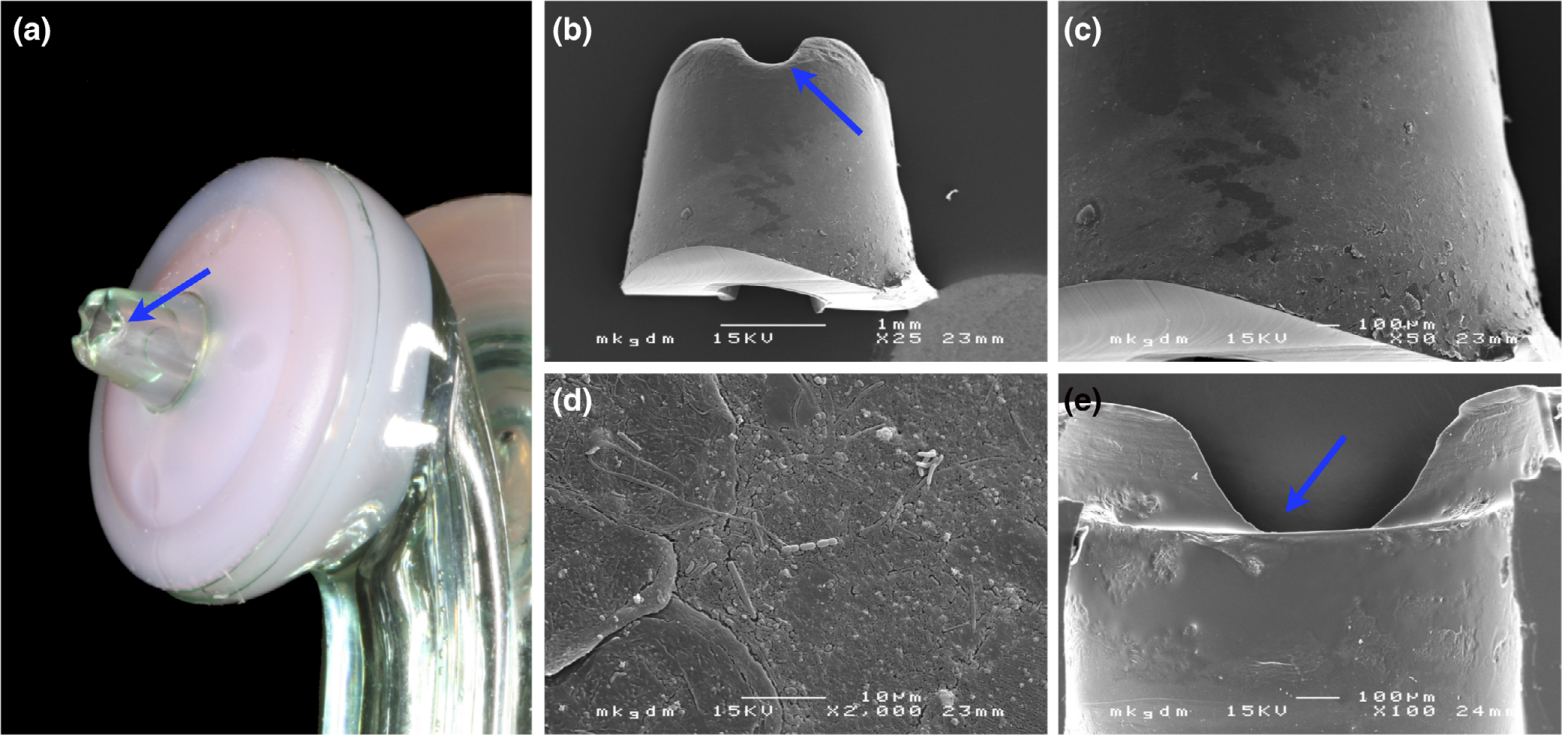
The tip of the nozzle of Participant #2 was examined by scanning electron microscopy. On the outside of the tip of the nozzle (a, b) a thin biofilm (c, d) was detected, but on the inside of the tip (e) biofilm formation was absent. The blue arrow (a, b, e) always indicates the same spot

SEM analysis of the tip of the nozzle of the device used by one of the participants (#2) presented a thin biofilm on the outside of the tip of the nozzle (i.e., at the aspect positioned into the interproximal space; Figure 2c,d), but no biofilm formation at the inside of the tip (Figure 2e). The bacterial deposit comprised of a variety of bacteria embedded in a very dense extracellular matrix sticking to the surface of the tip of the nozzle (Figure 2d).

## 4 DISCUSSION

The results of the present proof‐of‐principle study clearly showed that SAF devices are colonized by bacteria, not only oral species, even after a short period of regular use, and that these can be transmitted via the water‐jet. Specifically, both aerobic and anaerobic colonies were found in all water‐jet samples collected from all devices used intra‐orally (tests) and those used extra‐orally for 3 weeks (positive controls), while oral bacteria were found in the water‐jets of four out of five test devices.

Colonization with oral bacteria may come as no surprise, since the tip of the nozzle of the SAF comes in contact with the oral environment and colonization with oral bacteria has been previously reported for other intraoral cleaning devices such as toothbrushes (Ankola et al., 2009; Balappanavar et al., 2009; Frazelle & Munro, 2012; Mehta et al., 2007). Interestingly, positive control devices (i.e., devices used extra‐orally for 3 weeks) also showed contaminated water‐jet samples, but to a lower degree compared to the test devices and no colonization was seen with the tested oral bacteria. This finding, together with the fact that the water‐jet of negative control devices (i.e., brand‐new devices) as well as bottled water samples showed basically no contamination, indicates colonization of SAF also from extra‐oral sources, such as the skin, the cheek or fingers, or simply the direct surroundings, despite that the devices were used hygienically to prevent any contamination. Furthermore, the negative PCR results for all tested oral pathogens in Participant #3, despite the relatively high CFU count for aerobic and anaerobic bacteria, does not exclude the possibility of contamination from oral bacteria in this specific case; it is important to remember, that cariogenic bacteria, such as *Streptococcus mutans*, were not assessed in this study.

Since in SAF water is passed from the container of the device to the nozzle and tip, through a circuit of channels/pipes inside the device, it appears logical that biofilm formation in the nozzle and/or the pipework occurs also in SAF, similarly to every aqueous pipework (Gagnon et al., 2005; Wang et al., 2012). Nevertheless, it is noteworthy that this colonization of the nozzle and/or device results in a contaminated water‐jet delivered into the oral cavity. Thus, depending on the localization of the biofilm, that is, in the nozzle and/or in the device itself, the potential risk of cross‐contamination among family members/partners should be considered. Currently, it is recommended that one device may be used by more than one person and only the nozzle should be exchanged (i.e., each family member/partner should have its own nozzle). If the localization of the biofilm is not only in the nozzle, but also in the device itself, it is apparent that exchanging the nozzle may not be an adequate protection measure against cross‐contamination by oral and/or other pathogens. In perspective, colonization of the oral cavity through cross‐contamination, for example, from the mother to the child or between partners has been shown/suggested for cariogenic and periodontal pathogens (Berkowitz, 2006; Kort et al., 2014; Okada et al., 2004; Tamura et al., 2006). Nevertheless, it is uncertain, whether a daily limited/single exposure to a certain number of exogenous bacteria—as the one expected through the water‐jet of SAF—is actually sufficient to lead to a permanent change/manipulation of the oral microbiome of the exposed person. For example, it has been shown that even intimate kissing, which is assumed to cause an average transfer of 80 million bacteria within 10 s kiss duration, requires a relatively high daily frequency to result in some degree of shared salivary microbiota (Kort et al., 2014). However, the SAF should be also considered as a potential source for re‐infection during periodontal treatment. Thus, if the device was already used before initiating periodontal treatment, continuous use of the same nozzle and/or device—depending on the localization of the biofilm—may to a certain degree contribute to re‐infection of the treated periodontal pockets. In a similar fashion, several microbial niches in the oral cavity other than the pockets (e.g., tongue, tonsils) have been discussed as potential sources for re‐infection (Quirynen et al., 1995; Teughels et al., 2009).

The localization of this colonization (i.e., only in the nozzle or also in the device itself) was not within the scope of this proof‐of‐principle investigation; as sampling was performed only with a used nozzle, no assumptions can be made regarding the localization of this colonization. Lack of biofilm formation at the inside of the upper part of the nozzle (Figure 2), observed by SEM analysis in a single case in this study, may indeed indicate that colonization was only localized in the upper part of the nozzle and that the entire amount of the biofilm was transmitted (removed) with the water‐jet, that is, the nozzle gets cleaned through by the shear stress of the microburst of high velocity air and liquid micro‐droplets generated by the device, analogous to what is in theory happening at the teeth. However, as neither the entire nozzle nor the device itself was examined, bacterial colonization deeper inside the nozzle or within the device cannot be excluded. The fact that cases with a positive water‐jet result for a given oral pathogen, also presented relatively higher numbers of the specific bacterium in the saliva sample, may indicate some correlation between the health status (e.g., healthy person vs. periodontitis patient) and the relative risk for colonization of the SAF with periodontal pathogens; however, due to the limited number of participants herein, no final assumptions on this should be made.

Future studies should assess the localization of the biofilm, and the effect of specific cleaning procedures to reduce or prevent biofilm build‐up, as the currently recommended approach to empty and carefully swab the container with a clean paper tissue after each use is inefficient. For example, specific cleaning procedures (e.g., simple immersion in 0.12% chlorhexidine gluconate or 1% sodium hypochlorite for 20 h) have been described to reduce successfully the bacterial load on toothbrushes (Balappanavar et al., 2009; do Nascimento et al., 2014; Mehta et al., 2007; Nelson‐Filho et al., 2000, 2006). Furthermore, the possible impact of using SAF with a mouth‐rinse instead of water on the bacterial colonization of the nozzle and/or the device should be assessed, as the manufacturer in fact recommends using the SAF with tap water or with a mouth‐rinse. Finally, it has to be stressed that the findings of the present proof‐of‐principle study are not restricted to this specific water flosser, but most likely apply to all oral irrigators—since all come in contact with the oral environment and have a pipework system.

In conclusion, the present study showed that daily use of SAF for 3 weeks resulted in bacterial colonization in the nozzle and/or device with both aerobic and anaerobic, not only oral, species, that are transmitted via the water‐jet.

## CONFLICT OF INTEREST

All authors declare no conflict of interest.

## AUTHOR CONTRIBUTIONS

Kristina Bertl, Pia Edlund Johansson, and Andreas Stavropoulos: made substantial contribution in conception and design of the work, in the acquisition and analysis of data, and made substantial contribution in drafting and/or revising the work critically for important intellectual content. Corinna Bruckmann, Matthias Leonhard, and Julia R. Davies: have made substantial contribution in the acquisition and analysis of data for the work and in drafting the work. All authors approved the version to be published and agreed to be accountable for all aspects of the work.

## Data Availability

The data that support the findings of this study are available from the corresponding author upon reasonable request.
